# Morphological Plasticity and Phylogeny in a Monogenean Parasite Transferring between Wild and Reared Fish Populations

**DOI:** 10.1371/journal.pone.0062011

**Published:** 2013-04-19

**Authors:** Ivona Mladineo, Tanja Šegvić-Bubić, Rino Stanić, Yves Desdevises

**Affiliations:** 1 Institute of Oceanography & Fisheries, Split, Croatia; 2 Sardina d.o.o., Postira, Croatia; 3 UPMC Univ Paris 06, UMR 7232, BIOM, Observatoire Océanologique, Banyuls/Mer, France; 4 CNRS, UMR 7232, BIOM, Observatoire Océanologique, Banyuls/Mer, France; Thomas Jefferson University, United States of America

## Abstract

It is widely accepted that disease interactions between cultured and wild fish occur repeatedly, although reported cases have mainly relied just on the observation of similar symptoms in affected populations. Whether there is an explicit pathogen transfer between fish stocks, or each develops its own pathogen population, has been insufficiently studied and rarely supported by molecular tools. In this study, we used population dynamics and genetic structure of the monogenean *Furnestinia echeneis* in reared and neighbouring wild sea bream to indicate pathogen transfer, characterized by the phenotypic plasticity of the parasite attachment apparatus and the lack of phylogenetic differentiation. The observed pattern of genetic variation inferred by nuclear DNA Internal Transcribed Spacer 1 (ITS1) and mtDNA cytochrome C oxidase 1 (COI), between parasite populations is most likely caused by a recent shared demographic history like a reduced species area in the last glacial period. In spite of such recent expansion that populations underwent, *F. echeneis* shows differentiation in haptor morphometry as an adaptive trait in closely related populations at the aquaculture site. This suggests that differentiation in morphology may occur relatively rapidly in this species and that adaptive forces, not the speciation process, drives this monogenean parasitation. On the other hand, the observed phylogenetic inertia suggests a low to moderate gene flow (based on F*_ST_*) between parasites in cultured and wild fish, evidencing for the first time the transfer of pathogens at the aquaculture site inferred by a molecular tool.

## Introduction

As a response to an increasing demand for limited fish supply from fisheries, aquaculture has emerged as a high-throughput production of relatively cheap protein, significantly contributing to the enhancement of the global seafood offer to the market. Likewise aquaculture production estimated at around 45 million tons in 2004 has been predicted to reach 74 million tons by 2015 [1]. Such high-density intensive production system has also been regarded as an ideal milieu for pathogen transmission, amplification and spread of diseases to the surrounding environment [2]. However, since most of the pathogens causing diseases in farmed fish are also found in the wild cages-aggregating fish [3], cultured stocks, conditioned by demanding rearing pressure, may display more susceptibility to pathogens originating in the surrounding environment. This has been evidenced in a variety of geographical regions, and disease interactions between cultured and wild fish have been shown to occur repeatedly [3], [4], [5], [6]. Still, the effect of these interactions is poorly understood and there are conflicting views regarding their actual impact on the environment [7], [8]. While it is relatively easy to observe pathogen transfer if disease is contracted for the first time in the wild population after it has been diagnosed in introduced species, the evidence for potential continuous cross-contamination in natural conditions between two groups that share the same pathogen is more challenging [9], [10], [11]. Whether there is an explicit transfer of a given pathogen between wild and caged stocks, or each fish stock has been developing its own particular pathogen population, has been insufficiently studied and rarely supported by molecular tools (for a general review: http://www.dipnet.info/). The monogenean parasite *Furnestinia echeneis* (Diplectanidae) once forming the monospecies genus *Furnestinia* based on the distinctive feature of a single adhesive organ on the opistohaptor [12], has been transferred to genus *Lamellodiscus* based on the phylogenetic analysis of 18S and Internal Transcribed Spacer 1 (ITS1) rDNA [13]. Its adhesive organ, called lamellodisc, is composed of two vertical rows of lamellae and exists in duplicate in the genus *Lamellodiscus*, although less strongly developed. The parasite is strictly specialist for the sea bream *Sparus aurata* and has been recognised as a common and prevalent primary trigger in the course of secondary bacterial diseases in farmed bream in the Mediterranean [14]. As a haemophagous monogenean, it parasitizes gills, inflicting epithelial erosions and desquamations, necrosis, haemorrhages and secondary infections with ubiquitous bacteria during heavy infestations in warm season [15]. It is propagated directly by onocomiracidia that hatch from eggs after being laid by hermaphrodite parent, while morphogenesis in preadult larvae and adults occurs on fish gills. While in the past, Adriatic reared sea bream have been extensively parasitised by *Lamellodiscus elegans* [16], [17], only recently *F. echeneis* has been observed colonising its reared host [18], suggesting a high potential of this parasite to transfer from wild sea bream populations to reared fish.

In this paper, our goals were to a) define *F. echeneis* population genetic structure in reared sea bream (adult fish and newly seeded fingerlings) versus wild sea bream aggregating at the facility site, inferred from ITS1 rDNA and mtDNA cytochrome oxidase 1 (COI) markers, as well as potential differences in morphological features of the opistohaptor (lamellodisc); and b) estimate the length of time necessary for *F. echeneis* to colonised parasite-naïve fish (imported sea bream fingerlings) while at the same time identifying the origin of the colonising monogenean (wild *vs* reared adult fish).

## Materials and Methods

### Ethics Statement

This study was carried out in strict accordance with the recommendations of the Protocol for Work with Animal in Experiment of the Institute of Oceanography and Fisheries, Croatia and Regulation for protection of animals that are used in experiments or other scientific purposes of the Ministry of Agriculture, Fisheries and Rural Development of Croatia (NN 135/06). The study was approved by the Animal Ethics Committee of the Institute of Oceanography and Fisheries by a written permit (06/2009). Fish were sampled from the cages by hook and line and immediately exposed to excessive anesthesia by Eugenol®. When no opercular or fin movements were observed, fish were transferred to ice containers and transported to the laboratory. The same procedure was followed for wild fish, except that samples were collected by spear gun.

### 1. Fish Sampling

Cage reared sea bream (*Sparus aurata*) (N = 32) age +1, were sampled during summer months in 2010 from a single cage of a central Adriatic finfish facilities (Maslinova N 45°18′ E 16°27′). Monogeneans isolated from this sample were afterwards designated as population 1 - pop1. The average sea bream total weight was 119.82 g (±35.64 SD) and total length 19.82 cm (±9.13 SD). Wild sea bream (N = 22) were caught by hook and line or a spear gun at the same facility site, in close vicinity of sampled cage and measured 335.53 g (±146.03 SD) and 28.01 cm (±5.32 SD). Monogeneans isolated from this sample were afterwards designated as population 2 - pop2.

In the Gulf of Lion (Banyuls/Mer N 42°28′ E 3°8′ and Sète N 43°24′ E 3°40′, France), Mediterranean Sea, wild sea bream (N = 20) were caught by spear gun and measured 257.42 g (±63.23 SD) and 23.21 cm (±6.67 SD). Monogeneans isolated from this sample were afterwards designated as population 3 - pop3. In June 2010, imported sea bream fingerlings (N = 104; 4.66 g ±1.12 SD; 7.01 cm ±0.55 SD), were sampled at the arrival in the finfish facility (day 0; 3.67 g ±0.67 SD; 6.4 cm ±0.32 SD) till infection with monogenean *Furnestinia echeneis* was established (day 10; 6.32 g ±0.8 SD; 7.75 cm ±0.35 SD). Fish were killed by the overexposure of essential clove oil (Eugenol®) in concentration over 100 ppm. Parasitological examination was performed as previously described [16] and *F. echeneis* individuals (Monogenea, Polyopisthocotylea) were identified based on [19] and [20], respectively. The sample codes of parasites isolated from a specific host and geographic area that were sequenced in this study are given in Materials S1.

### 2. Opistohaptor Morphometry

Morphometry measurements were performed according to [21]. Briefly, at least 1–3 monogeneans were isolated from the gill arch of a single host, mounted on a slide with 2.5% sodium dodecyl sulphate to enable a clear visualization of the opisthaptor sclerotized parts. In total, ten monogeneans isolated from farmed fish and five from wild fish sampled in the vicinity of cages were used for measurement of opistohaptor morphometry. The same individuals were further fixed in absolute alcohol and stored at 4°C for phylogenetic analysis. A picture of the opistohaptor of each parasite was taken using a digital camera mounted on an Olympus CX41 light microscope, at 100× magnification. Images were loaded into DP-Soft software to perform measurements. Measurements were taken using landmarks method by taking distances between two points out of a total of eleven landmarks (Figure S1). For a specific landmark, minimum and maximum observed values were taken, subtracted and obtain value was divided by the designated number of morphotype classes (5). Further, for each landmark in respect to its measurement, a 0–4 class was assigned. Morphometry measurements (landmarks) were subjected to statistical multivariate techniques, using the STATISTICA software package (v. 7.0 for Windows), in order to discriminate morphotypes within *F. echeneis* individuals. First, to test whether or not the global morphometry of monogenean individuals differs between hosts, a principal component analysis (PCA) was conducted using all variables measured on the haptor. Also, each landmark value was compared between two types of host i.e. wild and reared seabream, using the Mann-Whitney test. Second, to define possible morphotypes, the Euclidian standardized morphometric distance between individuals of *F. echeneis* was computed using all measurements made on the haptor, and a clustering analysis was carried out, using the unweighted pair group method using arithmetic average (UPGMA) on all morphometric distances after standardization. Finally, linear discriminant analysis (LDA) was performed to verify the presence of pre-defined morphotypes.

### 3. DNA Isolation and Polymerase Chain Reaction (PCR), Product Sequencing and Alignment

For phylogenetic analysis, genomic DNA was isolated using the QIAGEN DNeasy Blood and Tissue Kit (Qiagen). Quantity and purity of isolated DNA and amplified fragments afterwards was checked by bio-photometer (Eppendorf). Each specimen DNA was used twice; for COI and ITS1 amplification, respectively.

A ∼900 bp fragment of Internal Transcribed Spacer 1 (ITS1) with partial 18S and 5.8S ribosomal DNA, was amplified using 0.8 µM of each primers: forward primer 5′ CAT CGT CGT GCC TGG GA 3′ and reverse primer 5′ GTA CAT AGA CAT CAC ACC AAG GT 3′. A mitochondrial DNA locus, COI (cytochrome C oxidase I) was amplified by PCR using 0.8 µM of each primers: forward primer 5′ GAG CTA AGT AAA AAT CAA GAA CC 3′ and reverse primer 5′ TCT ATC TAA CAC TGA GGC TG 3′, amplifying ∼300 bp of *F. echeneis* COI. The rest of the reaction mix for both markers consisted of DreamTaq buffer (Fermentas), 2 mM of MgCl_2_, 2 mM of each dNTP, 1.25 U of DreamTaq DNA polymerase (Fermentas) and 3 ng/µl of template. For both fragments the amplification profile consisted of initial denaturation for 3 min at 95°C, 35 cycles of denaturation for 30 sec each at 95°C, annealing at 56°C for 30 sec, elongation for 60 sec at 72°C with final extension of 10 min at 72°C. Products were loaded on a 1% agarose gel and visualized by adding SYBR Safe (1%) directly in the gel.

PCR products were purified using QIAquick PCR Purification Kit (Qiagen) and sequenced on an ABI 3100 automatic DNA sequencer (Applied Biosystems), using the ABI PRISM BigDye Terminator Cycle Sequencing Kit, in both directions. Sequences were aligned by Clustal X, implemented in the MEGA 5 software, using default parameters, and trimmed using GBlocks tool (http://molevol.cmima.csic.es/castresana/Gblocks.html).

### 4. Data Analyses

#### Parasite population dynamic

Parasites were counted on each gill arch and prevalence and abundance were calculated according to [22]. Sterne’s exact 95% confidence limits (CL) were calculated for prevalences, bootstrap 95% confidence limits (number of bootstrap replications  = 2000) for mean abundances, and exponent of the negative binomial (k) for the parasite skewness, using Quantitative Parasitology 3.0 software (QP3.0) [23]. Since parasites typically exhibit an aggregated (right-skewed) distribution within a host population, the negative binomial model represents the observed data following the maximum-likelihood method [24].

To test whether the mean abundance and intensity of parasites differs among three sea bream categories (wild, +1 year reared, fingerlings), bootstrap t-test was used, while differences among prevalences were tested by unconditional tests, both incorporated within QP3.0.

### Genetic Diversity and Population Structure

Nucleotide sequences of COI and ITS1 genes were compiled and aligned with Clustal X 1.83 [25], using default parameters and further verified by GBlocks. Molecular diversity was measured using Dnasp 5.0 [26] and Arlequin 3.5 [27]. Values for the number of haplotypes (H), polymorphic sites (S), haplotype diversity (h; [28]), nucleotide diversity (p; [28]) and the average numbers of pairwise nucleotide differences (k, [29]) were estimated. Pairwise and overall distances among haplotype sequences were calculated in MEGA 5 [30].

A substitution model for both genes and the gamma distribution shape parameter for the rate of heterogeneity among sites were determined using Modeltest 3.07 [31] based on the Hierarchical Likelihood Ratio Tests (hLRTs). For COX data, the TrN model [32] of evolution with the gamma shape parameter was selected for the analysis of molecular variances (AMOVA) and phylogenetic analysis. For ITS data, the JC model [33] of evolution with equal base frequencies was selected for the phylogenetic analysis.

For evaluation of hypothesized patterns of spatial genetic structure, a hierarchical analysis of molecular variance (AMOVA) was used to partition variance components attributable to population variance and to individuals within the populations, where 10000 permutations were performed to test the significance of pairwise population comparison. Pairwise genetic differentiation between populations was estimated using the fixation index F_ST_ and statistical significances were tested with 10000 permutations. The number of migrants per generation (*Nm*) between localities, AMOVA and F_ST_ calculations were performed in Arlequin 3.5. The null hypothesis of population panmixia was also tested in Arlequin 3.5 using an exact test of the differentiation of haplotypes among populations.

Tajima’s D [34] and Fu’s Fs [35] statistics were calculated to verify the null hypothesis of selectivity neutrality in relation to mtDNA sequences, which would be expected with population expansion.

Mismatch distributions [36] were constructed using Arlequin 3.5. The shapes of the mismatch distributions were used to deduce whether a population has undergone sudden population expansion [37]. The fit between the observed and expected distribution was tested using the Harpending raggedness index (Hri; [36]) and sum of squared deviations (SSD) for the estimated stepwise expansion models [38], implemented in Arlequin 3.5. Significance was assessed on the parameters with permutation tests under the null hypothesis that sudden population expansion cannot be rejected.

#### Phylogenetic analysis

Maximum likelihood (ML) analyses were performed on molecular data sets, which included concatenated sequences from both genes. The ML heuristic searches were performed in PAUP* v4.0b10 [39], using Hasegawa, Kishino and Yano 1985 (HKY85+g) model of nucleotide substitution and parameter values selected via Modeltest. Bootstrap resampling was conducted using 1000 replicate neighbour-joining (NJ) trees based on the ML substitution matrix. Prior ML analysis, partition homogeneity test using 100 random partitions of the entire data set was performed to determine if two gene regions exhibited significantly different phylogenetic signal.

Concatenated phylogenetic tree was constructed from the tree output file produced in the maximum likelihood analysis and visualized using FigTree (http://tree.bio.ed.ac.uk/software/figtree/).

Finally, to illustrate haplotype distribution among sampled groups, a median-joining network [40] was inferred using only COI haplotypes and the program NETWORK v 4.5.1.6 (available at http://www.fluxes-engineering com/sharenet.htm).

## Results

### 1. Population Dynamic of Furnestinia Echeneis

The prevalence of *F. echeneis* in the wild sea bream population (N = 22) was 71.1% (95% confidence limits 69.32–79.51), intensity 3.24 (bootstrap 95% confidence limits 2.82–3.63) and abundance 2.24 (bootstrap 95% confidence limits 1.85–2.92) parasite per fish. In cage-reared +1 year old sea bream (N = 32), prevalence was 73.2% (95% confidence limits 61.54–78.12), intensity 3.95 (bootstrap 95% confidence limits 3.48–4.35) and abundance 2.73 (bootstrap 95% confidence limits 2.09–3.24) parasites per fish. In imported fingerlings (N = 104), *F. echeneis* oncomiracidia were observed on the 10th day after seeding in semi offshore cages, with prevalence of 20% (95% confidence limits 18.17–23.33), intensity 1.54 (bootstrap 95% confidence limits 1.32–1.78) and abundance of 0.2 (bootstrap 95% confidence limits 0.18–0.23) parasites per fish.

The smallest infected sea bream (fingerling) measured 7.5 cm and 6.28 g, while the largest one measured 32 cm and 448.15 g, respectively. Monogeneans were aggregated across host populations (right biased), and most sea breams harboured low numbers of parasites. The greatest number of parasites (N  = 14) has been harboured by a male of 23 cm and 219.87 g. Fitting the negative binomial distribution as a theoretical model to the observed data following the maximum-likelihood method, observed and expected frequencies did not differ significantly (χ^2^ = 359.99, p  = 0.05), with exponent of the negative binomial k  = 0.828 (reared fish); 1.021 (wild fish); 0.07 (fingerlings), indicating non-random contact of *Furnestina* and sea bream.

Comparing prevalence, abundance and intensity values between fish categories, no difference was observed between wild and +1 year old reared sea bream, while a significant difference between adult fish categories and fingerlings was noticed (*P*<0.05).

### 2. F. Echeneis Morphotype Diversity

**Figure 1:**
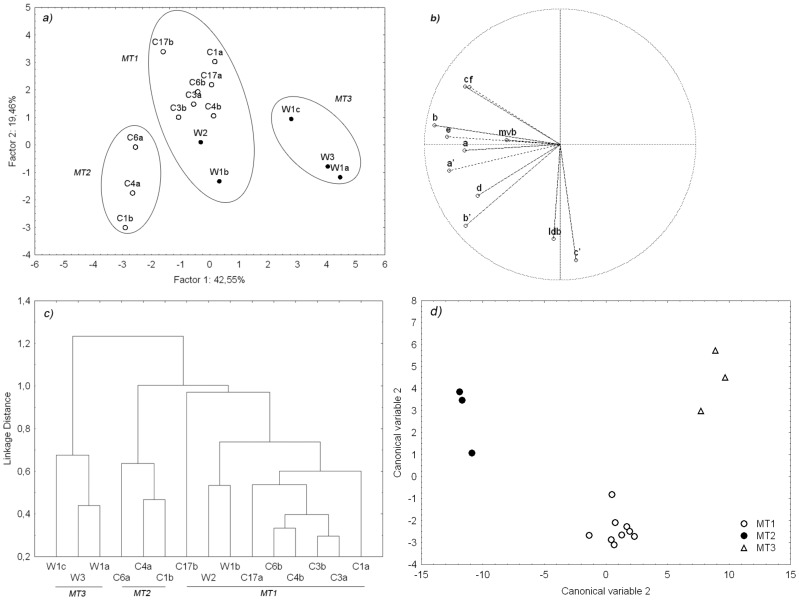
Principal component diagram (Factor 1 and Factor 2) of all morphometric variables measured on *Furnestinia echeneis* parasites from *Sparus aurata* from the Adriatic Sea. Solid circles indicate the tree different morphotype groups observed. Each parasite is identified by a letter and number corresponding to the host origin (a); Correlation diagram displaying variables in the two-factor space (PC1 vs. PC2) (b); dendrogram reconstructed using UPGMA on the Euclidian distances computed from standardised morphometric variables between parasite individuals from *S. aurata* with outlined morphotype groups (c); ordination diagram showing results of the discriminant analysis in the space defined by first two discriminant functions (canonical variables). Three main morphotype groups are clearly separated (d)., cage Adriatic population; •, wild Adriatic population; MT1, morphotype 1; MT2, morphotype 2; MT3, morphotype 3.

Analysis of space formed by first and second principal components, denoted as factor 1 and factor 2, showed that the majority of the parasites from the Adriatic cage and Adriatic wild sea breams are grouped and form the homogenous central part of the score plot (Fig. 1a). Three individuals from the wild sea breams and three from cage sea breams are clearly discriminated, weighting positively first PC for first group, and negatively for the second one. We were able to discern three provisional, well defined, morphotype groups (marked MP 1–3), supported by the dendogram built from Euclidian standardised morphometric distance (Fig. 1c).

According to the elemental projection plot (Fig. 1b), it can be inferred that variances between the groups are mainly controlled by fluctuations in length of hamuli (a, a’), point length (b, b’), hook opening (c, c’), root length (e) and guard length (f). It must be noted that afore mentioned morphometric values were all significantly larger in individuals from cage fish, in comparison to the individuals from wild fish (Mann-Whitney test, *P*<0.05). The contribution of median ventral bar (mvb) and lateral dorsal bar (ldb) to variances between groups was negligible.

The LDA classification was performed in order to test for three pre-defined morphotype groups based on their morphological similarity. Using the log-transformed variables, the LDA correctly classified 100% of the cases. Variables having the greatest statistical significance (*F*-factor in LDA ≥7.00) in discrimination between the groups are c’, b’, and f. The discriminant function 1 accounts for 60.42% of total variance. A bivariate plot of CV1 versus CV2, shown in Figure 1d, illustrates the segregation of the morphotype groups (MT1-3) clearly showing morphometric homogeneity within groups.

### 3. Genetic Diversity of the COI and ITS1 Gene Fragments

Unambiguous sequences for the mitochondrial COI and nuclear ITS1 genes of three *F. echeneis* populations from different geographic origins were obtained and deposited in GenBank (JX089988-JX090041 for COI locus and JX090045-JX090099 for ITS1 locus). The length of the COI and ITS1 fragments used in the analysis was 279 bp (n = 66) and 969 bp (n = 55), respectively. In total, 20 variable sites were observed and 17 haplotypes were detected for COI fragment while for ITS1 fragment, 19 variable sites and 9 haplotypes were detected (Results S1, S2). Sequence divergence (Tamura and Nei distance) among COI haplotypes ranged from 0.05% to 1.14%, with an average of 0.26%, while among ITS1 haplotypes (Jukes-Cantor distance) ranged from 0.05% to 1.38%, with an average of 0.32%. Among 20 polymorphic sites observed in COI fragment, 11 were singleton variable sites and 9 were parsimony informative. Among 17 haplotypes defined, most (65%) were unique and represented by a single individual. Only three were shared among sampled localities (H1, H8, H13) and H1, the most common haplotype, was highly present in both Adriatic Sea populations. Among 19 polymorphic sites observed in ITS1 fragment, 14 were singleton variable sites and only 5 were parsimony informative. Among 9 haplotypes defined, 78% were unique and represented by a single individual. Only two were shared among sampled localities (H1 and H2) while H1, the most common haplotype, was present in all localities.

Genetic diversity indices for each population are summarised in Results S3 and S4, giving an overall haplotype diversity (h) of 0.749±0.054 and nucleotide diversity (π) of 0.0088±0.0011 for COI fragment, indicating high levels of haplotype diversity and low nucleotide diversity. For ITS1, haplotype diversity was less expressed, reaching the overall value of 0.359±0.082, followed by low nucleotide diversity of 0.0014±0.0005. As well, COI displayed higher value of average number of nucleotide differences (2.4545±1.34) compared to ITS1 fragment (1.2365±1.35).

### 4. Population Genetic Structure

Estimates of pairwise genetic differentiation (F*_ST_*) and gene flow (*Nm*) among population are shown in Table 1. For both gene fragments, global F*_ST_* values were generally high, showing significant genetic structure in the range investigated (F*_ST_*(COI)  = 0.60, P<0.001; F*_ST_*(ITS1)  = 0.29, P<0.001). Pairwise F*_ST_* values among Adriatic and Mediterranean populations were high and significant, whilst among wild and reared Adriatic populations F*_ST_* values were low and not significant, leading to much higher *Nm* estimated values in comparison to the Adriatic-Mediterranean values (Table 1). Genetic structure was also confirmed by AMOVA based on COI fragment, which attributed 60% of the genetic variation to variability among population, and 40% variation within populations. For less divergent ITS1 region, AMOVA revealed that 28.7% of the genetic variation occurred among populations, whereas 71.3% of the genetic variation occurred within populations (Results S5). Over all samples, non-differentiation exact P values were significant (P  = 0.00) for both genes, rejecting that the population of *F. echeneis* along investigated area is panmictic.

**Table 1:** Estimates of pairwise genetic differentiation (*F*_ST_) and gene flow (*Nm*) among populations of *Furnestinia echeneis* based on mitochondrial COI (lower diagonal) and ITS1 (upper diagonal, in italic) sequences data.

	Pop1	Pop2	Pop3
Pop1		0.001 (419)	0.403* (0.74)
Pop2	−0.022 (inf)		0.290* (1.22)
Pop3	0.692* (0.22)	0.661* (0.25)	

In parentheses: Nm values; significance was tested with 10000 permutations, with significantly different populations indicated with an asterisk.

### 5. Phylogenetic and Network Analyses

**Figure 2:**
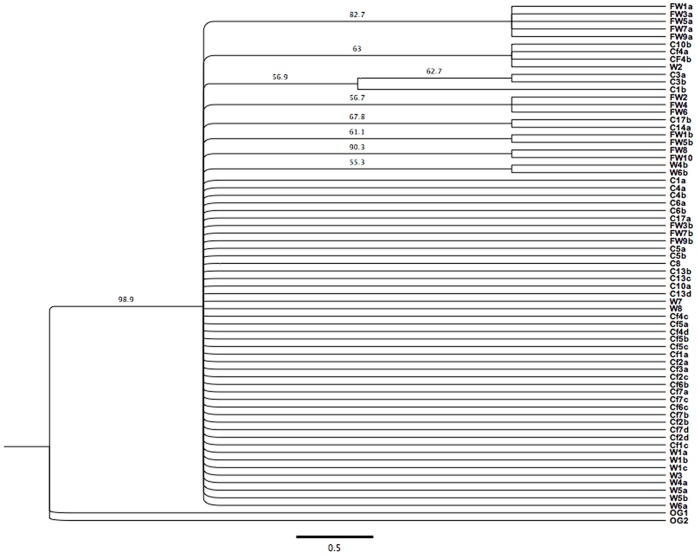
Concatenated phylogenetic tree (COI and ITS1) inferred by maximum likelihood (ML) analysis of *Furnestinia echeneis*. C - cage, Cf - cage fingerlings; W - wild; FW - France wild; OG1 - outgroup 1 *Lamellodiscus ignoratus* (JF427655); OG2 - outgroup 2 *L. ergensi* (JF427653.1) numbers at the end of sequence codes stand for fish numbers; small alphabetic letters (*a*, *b*, *c*, *d*) stand for monogenean individuals isolated from the same fish.

**Figure 3:**
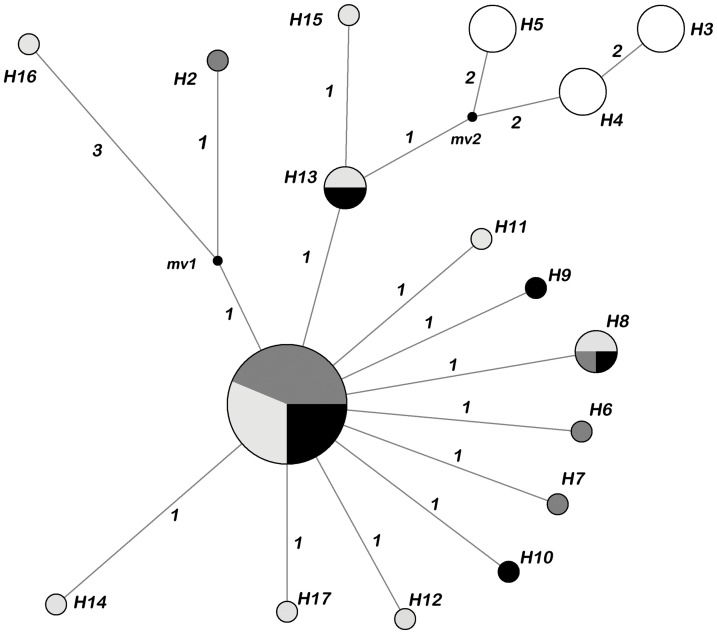
Median-Joining network showing the relationships among *Furnestinia echeneis* haplotypes defined by COI sequences variation. Numbers along each branch designate the number of base differences among haplotypes. Circle sizes are proportional to the frequency of each haplotype. mv1–mv2 are median vectors and represent the possible extent of unsampled sequences or extinct ancestral sequences. In black, grey, white-grey and white background, *F. echeneis* haplotypes sampled from Adriatic wild sea bream, Adriatic cage sea bream adults, Adriatic cage sea bream fingerlings and western Mediterranean wild sea bream (Gulf of Lion), respectively.

The topology of tree built from concatenated sequences of two genes using maximum likelihood (ML) (Fig. 2) was shallow and unresolved, missing well-supported groups. Populations were scattered throughout the tree and the clustering was mainly observed for French wild population. The haplotype network showed a star-like phylogeny with most of the unique haplotypes closely related to the common central haplotype (H1), with exception of Mediterranean very divergent haplotypes (H3, H4, H5), evident also from the geographical structure (Fig. 3). Central haplotype was composed from cage adults/cage fingerlings/wild Adriatic populations. If *F. echeneis* underwent expansion, the central common haplotype was likely the ancestral one, from which one or a few stepwise nucleotide substitutions could explain all other unique haplotypes.

### 6. Demographic Patterns

The demographic history of *F. echeneis* was investigated using mismatch distribution. For both genes, the goodness of fit test showed that no mismatch distributions for sample localities deviated significantly (*P*>0.05) from predicted values under the sudden expansion model of [37] (Results S6 and S7; Figure S2 and S3). Furthermore, the HRI values were low for Adriatic and overall populations, indicating a significant fit between the observed and the expected distribution, thereby providing further evidence for population expansion in the past [36]. Only Harpending’s raggedness index for the western Mediterranean population was significant but sum of squared deviation for this group did not support a departure from the null hypothesis of population expansion. The θ-values for all cases confirmed the scenario of population expansions, the initial θ-values were always much smaller than the final θ-values (Results S6 and S7). Departure of the age expansion parameter (τ) between Adriatic populations and western Mediterranean population, observed in both genes, suggest that, for these areas, the population expansion may date back to different historical period.

The results of Tajima’s D test and Fu’s F_S_ test are presented in Results S6 and S7. Tajima’s D values were negative for Adriatic and overall populations, indicating an excess of rare nucleotide site variants compared to the expectation under a neutral model of evolution. Only for the pop1 population in COI region and pop1, pop2 and overall populations in ITS1 region, these deviations from neutrality were significant. Fu’s F_S_ test, which is based on the distribution of haplotypes, also showed negative values for Adriatic and overall populations, indicating an excess of rare haplotypes over what would be expected under neutrality. Following this test, the hypothesis of neutral evolution was rejected for all populations except for the western Mediterranean population (pop3).

## Discussion

*Furnestinia echeneis* population dynamics between wild and reared fish are the same, indicating that the same ecological traits work simultaneously on both sides of the net barrier. In the wild environment fish would not show such elevated values of parasite population, since host interactions, food availability and other ecological factors are far less pronounced than for agglomerated enclosed fish hosts in aquaculture, where parasite proliferation is supported by high density, stress, and organic load. However, since farming facilities are known to represent fish aggregating devices (FAD), similar ecological factors within cages can be drawn to fish populations aggregating in their vicinity, resulting in the same dynamic values for *F. echeneis* in this case. Although we have not assessed *Furnestinia* dynamic through 1-year sampling, only in its most proliferative period (summer months), the same values have been observed in previous study [18] and we suggest that they depict a good representation of the monogenean dynamic.

Analysing population genetic structure of the monogenean *F. echeneis* infecting Mediterranean and Adriatic sea bream, we have evidenced the existence of genetically highly structured populations. Such genetic structuring of all three studied *F. echeneis* populations could be attributed to parasite ecological characteristics as wide distributional range (Adriatic vs. Gulf of Lion), the fragmented nature of the habitat or/and low expected rate of long distance dispersal [41]. Asexual hermaphrodite type of reproductive found in this monogenean might also limit the possibility of the long-distance gene flow, fixing the different alleles in different subpopulations and making them more structured. However, such structuring showed an exception only in the case of parasite population infecting caged fish (pop1) and wild specimens in their proximity (pop2), suggesting a constant gene flow and pathogen transfer. Populations pop1 and pop 2 are especially closely related to each other as indicated by a very low and non-significant F_ST_ value. To our knowledge, this is the first molecular evidence of pathogen transfer between fish in aquaculture and in the wild. Genetic differentiation of sea louse *Lepeophtheirus salmonis* infecting farmed and wild salmon has been previously studied using allozyme polymorphism and RAPD [42]. The authors showed polymorphism, but failed to obtain consistent results, because allele frequency analysis inferred by allozymes suggested no evidence of genetic differentiation between farm and wild salmon, whereas RAPD showed a clear distinction. Only at a single sampled farm, RAPD phenotypes were closely similar to the wild-type sea lice. Such discrepancies might have been influenced by the very nature and uniformity of the farm environment that itself provides selection pressure [42], as well as representativeness of the samples and sensibilities of techniques used (see [43]).

*Furnestinia* molecular diversity indices showed to be similar between the populations for the two analysed loci, although in both cases monogeneans from reared fish showed higher diversity indices that might have been expected due to their potential isolation in the cages. Haplotype diversity lay in the range 0.60–1 (COI), which is high in comparison to many other parasite species. Similar high haplotype diversity values have been reported in another monogenean species, *Mazocraeoides gonialosae* (Monogenea, Mazocraeidae) [44]. Although haplotype diversity was high, low nucleotide diversity values indicated only small differences between haplotypes, and this was also evident from concatenated phylogenetic tree. Maximum likelihood analysis revealed relatively shallow and unresolved paraphyletic lineages, with no significant genealogical branching or clusters of corresponding morphotypes. Such combination of high haplotype diversity and low nucleotide diversity as observed in our data can be a signature of a rapid demographic expansion from a small effective population size [45].

Recent population expansion is suggested for the Adriatic populations of *F. echeneis* based on Tajima’s D and Fu tests. A positive value would have indicated a bias towards intermediate frequency alleles, while a negative value would have indicated a bias towards rare alleles, the latter being a landmark of recent population expansion. This further suggests a relatively recent colonisation event of the Adriatic host. In consequence, Mediterranean and Adriatic *Furnestinia* populations showed incongruence in their expansion time, the former having more ancient population expansion pattern. Alternatively, negative values of Tajima and Fu’s tests could have been the outcome of balancing selection at a nearby locus, although studies demonstrating direct or indirect selection (through hitchhiking) on the mitochondrial genome in natural populations are rare to point a general rule [46]. This was the reason why, in addition to a mitochondrial locus, we included in the analysis a neutral nuclear DNA marker to obtain a more complete perspective on the neutral population structure of the populations. The explanation of recent demographic expansion in *Furnestinia* populations fits with the widely observed pattern of population expansion in organisms across taxa following the last glacial period, which ended around 12,500 years ago.

*F. echeneis* experienced a transfer from wild to aquaculture fish, and as an hermaphroditic, specialist and fast-reproducing parasite with direct life-cycle, it generated a high diversity of haplotypes and discrete populations within Adriatic area. Haplotypes divergence (COI 0.016 to 2.336%; ITS1 0.055 to 0.749%) is in accordance with previous data for monogenean species: *Haliotrema aurigae* (0.17–0.7%), *Euryhaliotrematoides grandis* (0.17–7.99%), *Polylabroides* (0.12–1.89%) and *Microcotyle* (0.14–2.56%) [44]. Moreover, our data do show that only a small proportion of haplotypes has been shared between wild and reared fish, while the majority of parasites show unique haplotypes. A high percent of unique haplotypes for COI and ITS1 (70% and 78%, respectively) reflects a high genetic diversity of the parasite even within geographically limited area (aquaculture facilities). It seems that the transfer of those few rare haplotypes that succeeded to exchange between wild/reared fish populations, has been influenced by a scarce number of interactions between reared and wild fish, a fact that generally has been thought as unlikely in the site of high host density. This is also supported by the estimation of wild sea bream abundance aggregating near aquaculture sites. Wild fish aggregations in the vicinity of aquaculture cages have been recently studied in Spain, revealing a very high number of species recorded, with two families, Sparidae (12 species) and Carangidae (4 species) being particularly abundant [47]. Among sparids, the abundance of wild sea bream is influenced by farm location and season, but around Spanish fish farms, it seems to be considerably lower [48] compared to Croatian sampling site (Stanić, personal comm). This greatly affects the chances for pathogen transfer and consequently the number of haplotypes shared between *Furnestinia* populations.

Colonization of the monogenean-free fingerlings that originated from a biosecurity hatchery systems, by oncomiracidia, has been evidenced to occur relatively fast (10th day after transfer in cages) and in all cases, oncomiracidian haplotypes were shared with *Furnestinia* haplotypes parasitising adult reared sea bream. Furthermore, the haplotype network suggests that the most recent haplotypes belong to monogeneans isolated from fingerlings, and are divergent from the most abundant haplotype 1. This supports the hypothesis that pathogen transfer from adult caged fish to fingerlings has more probability to occur than the one from wild aggregating fish, emphasising the importance of widely neglected zootechnical measure that culturing sites, with sea currents taken in consideration, have to be designed in order to separate reared fish in different age categories. Differences in *Furnestinia* abundances between reared sea bream adults and fingerlings is attributed solely to the short period of fingerling sampling (e.g. as soon as the parasite has been established on the gills the sampling was stopped), and given enough time, *Furnestinia* would equalise its abundances in adult and fingerling hosts.

Another characteristic of the *Furnestinia* population is that it does not show evidence of neutral evolution [49] as the vast majority of evolutionary changes at the molecular level are likely not being caused by random drift of selectively neutral mutants, but adaptive changes. Such positive selection is further supported by haptor morphometry, evidencing that the most abundant haplotypes are the one represented by longer hooks (landmarks a and b, respectively) of the haptor. Haptor, via its morphology and strength for attachment, is an important character for survival of monogeneans on their hosts: it is necessary for the parasite to hold the grip on gills in order to avoid to being expelled by water currents in the gill chamber. Its variability is more pronounced in generalist species and it has been suggested that variations in the length of different parts of the organ could be considered as a by-product of adaptation to the host [21], [50]. Such phenotypic plasticity has been already observed in other monogeneans [51], [52]. Unambiguously we have evidence supportinga major difference in haptoral elements between neighbouring wild and reared fish, with the later having more pronounced increase in size. In the genus *Lamellodiscus*, it is not unusual that the description of new species is documented as morphological variants of previously described species [53], leading to the questionable erection of new species based on indiscrete small changes rather than assignment of such individuals to morphotypes [21]. In generalist *Lamellodiscus* species, unlike *Furnestinia*, previous authors suggested that morphometric differences would indicate radiation and further speciation. However, in *Furnestinia* populations, shared by two closely sympatric fish populations, longer haptoral components in parasites from reared fish may represent a rapid adaptation to the local environment where excessive gill mucus production [54], usually associated with rearing conditions, could trigger such adaptation. Gill mucus in fish serves as a mechanical vehicle to wash off alien elements, as well as a prime site of activity of innate immune system [55], [56]. Its more abundant secretion in cultured fish imposes unequivocally more pressure on the parasite. Likewise, in cultured sea bream infected with copepod *Ergasilus sieboldi*, mucus cells encircling the parasite were significantly more abundant in infected than uninfected tissue [57], which, along with immunological properties of gill mucus, suggests active involvement in parasite rejection. We suggest that such host mechanism of increased mucus secretion induces changes to longer haptoral elements in *Furnestinia* infecting reared sea bream compared to wild fish. Such phenotypic plasticity in monogenean sclerotized parts, unsubstantiated with genetic differentiation between parasite population in reared and wild fish, could be also induced by other environmental factors like temperature [58], but the synchronous collection of sympatric samples that we performed rules out such possibility.

In conclusion, observed patterns of genetic variation within and between *Furnestinia* populations are most likely caused by a recent demographic history in the form of a reduced species area in the last glacial period. In spite of such recent expansion, this monogenean shows differentiation in haptor morphometry in closely related populations at the aquaculture site, suggesting that differentiation in morphology and potentially in other traits may occur relatively rapidly in *F. echeneis* and that adaptive forces, not the speciation process, drives this monogenean parasitation. On the other hand, the observed phylogenetic structure suggests a free gene flow between parasites in cultured and wild fish, providing evidence for the transfer of pathogens at the aquaculture site inferred by a molecular tool.

## Supporting Information

Figure S1 **Morphometric variables (landmarks) measured on the sclerified parts of the opisthohaptor of** ***Furnestinia echeneis*** **individuals: a, a’ - total length of dorsal and ventral hook; b, b’ - point length of dorsal and ventral hook; c, c’ - blade opening of dorsal and ventral hook; d - distance between grip and hilt; e - grip length; f - hilt length; mvb - median ventral bar; ldb - lateral dorsal bar.**
(TIF)

Figure S2 **Mismatch distribution of cytochrome c oxidase I (COI) haplotypes of** ***Furnestinia echeneis*** **sampled from Adriatic wild sea bream (a), Adriatic cage sea bream (b) and western Mediterranean wild sea bream (c).**
(TIF)

Figure S3 **Mismatch distribution of internal transcribed spacer 1 (ITS1) haplotypes of** ***Furnestinia echeneis*** **sampled from Adriatic wild sea bream (a), Adriatic cage sea bream (b) and western Mediterranean wild sea bream (c).**
(TIF)

Materials S1 ***Furnestinia echeneis*** **samples, with geographic area of sampling, source, host category and allocated GenBank accession numbers.**
(DOC)

Results S1 **Number of COI haplotypes obtained in each population and changes in base composition.**
(DOC)

Results S2 **Number of ITS1 haplotypes obtained in each population with changes in base composition.**
(DOC)

Results S3 **Sampling localities, hosts and descriptive statistics of genetic diversity of** ***Furnestinia echeneis*** **, based on COI sequence data.**
(DOC)

Results S4 **Sampling localities, hosts and descriptive statistics of genetic diversity of** ***Furnestinia echeneis*** **, based on ITS1 sequence data.**
(DOC)

Results S5 **Analysis of molecular variance (AMOVA) for** ***Furnestinia echeneis*** **populations, based on mitochondrial COI and ITS1 (in italic) sequences data.**
(DOC)

Results S6 **Tajima's** ***D*** **, Fu's** ***Fs*** **statistics, corresponding** ***P*** **values and mismatch distribution parameter estimates for** ***Furnestinia echeneis*** **based on COI sequence data.**
(DOC)

Results S7 **Tajima's** ***D*** **, Fu's** ***Fs*** **statistics, corresponding** ***P*** **values and mismatch distribution parameter estimates for** ***Furnestinia echeneis*** **based on ITS1 sequence data.**
(DOC)
