# Supplementary figures and images for: Morphological Plasticity and Phylogeny in a Monogenean Parasite Transferring between Wild and Reared Fish Populations
Source: PLoS One. 2013 Apr 19;8(4):e62011. doi: 10.1371/journal.pone.0062011 (PMC3631154; doi:10.1371/journal.pone.0062011)

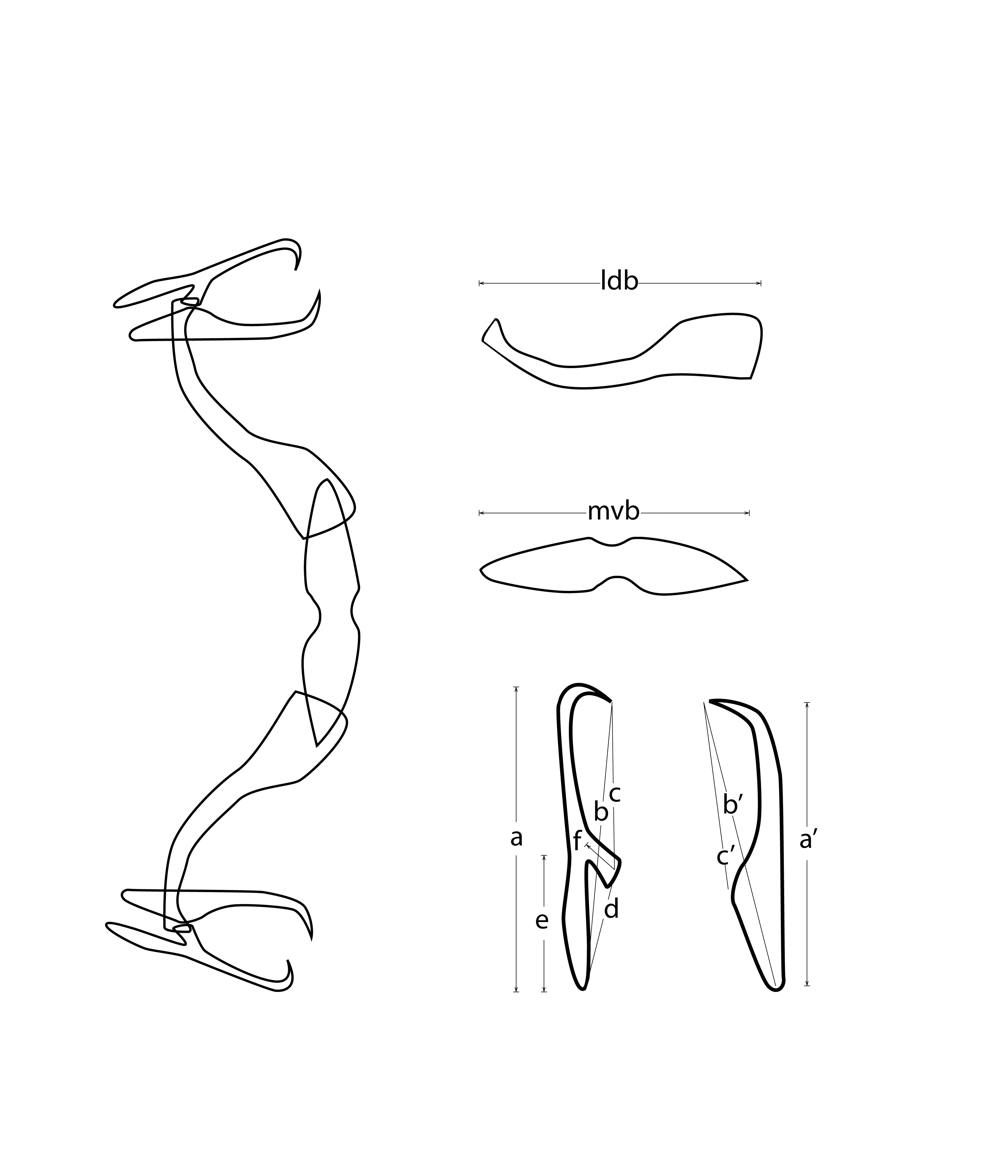

Supplement: Figure S1 — Morphometric variables (landmarks) measured on the sclerified parts of the opisthohaptor of Furnestinia echeneis individuals: a, a’ - total length of dorsal and ventral hook; b, b’ - point length of dorsal and ventral hook; c, c’ - blade opening of dorsal and ventral hook; d - distance between grip and hilt; e - grip length; f - hilt length; mvb - median ventral bar; ldb - lateral dorsal bar. (TIF) [file pone.0062011.s001.tif]

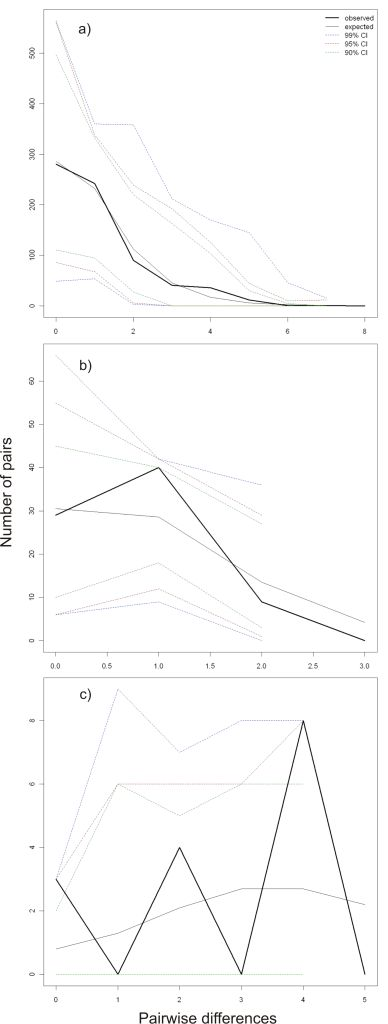

Supplement: Figure S2 — Mismatch distribution of cytochrome c oxidase I (COI) haplotypes of Furnestinia echeneis sampled from Adriatic wild sea bream (a), Adriatic cage sea bream (b) and western Mediterranean wild sea bream (c). (TIF) [file pone.0062011.s002.tif]

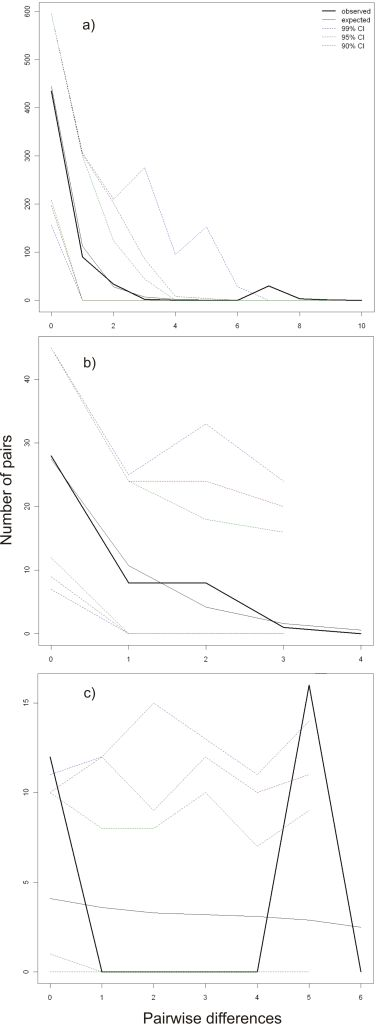

Supplement: Figure S3 — Mismatch distribution of internal transcribed spacer 1 (ITS1) haplotypes of Furnestinia echeneis sampled from Adriatic wild sea bream (a), Adriatic cage sea bream (b) and western Mediterranean wild sea bream (c). (TIF) [file pone.0062011.s003.tif]
