# Supplementary material for: Morphological Plasticity and Phylogeny in a Monogenean Parasite Transferring between Wild and Reared Fish Populations
Source: PLoS One. 2013 Apr 19;8(4):e62011. doi: 10.1371/journal.pone.0062011 (PMC3631154; doi:10.1371/journal.pone.0062011)
Supplement: Materials S1 — Furnestinia echeneis samples, with geographic area of sampling, source, host category and allocated GenBank accession numbers. (DOC) [file pone.0062011.s004.doc]

|  |  |  |  |  |  |
| --- | --- | --- | --- | --- | --- |
| Sample code | Geographic area | Source | Host | GenBank COI | GenBank ITS1 |
| C1a | Adriatic | cage | adult | JX089988 | JX090055 |
| C1b | Adriatic | cage | adult | JX089989 | JX090056 |
| C3a | Adriatic | cage | adult | JX089990 | N/S |
| C3b | Adriatic | cage | adult | JX089991 | JX090057 |
| C4a | Adriatic | cage | adult | JX089992 | JX090058 |
| C4b | Adriatic | cage | adult | JX089993 | JX090059 |
| C5a | Adriatic | cage | adult | JX090001 | JX090063 |
| C5b | Adriatic | cage | adult | JX090002 | JX090064 |
| C6a | Adriatic | cage | adult | JX089994 | JX090060 |
| C6b | Adriatic | cage | adult | JX089995 | N/S |
| C8 | Adriatic | cage | adult | JX090003 | JX090065 |
| C10a | Adriatic | cage | adult | JX090006 | JX090067 |
| C10b | Adriatic | cage | adult | JX090007 | JX090068 |
| C13b | Adriatic | cage | adult | JX090004 | JX090066 |
| C13c | Adriatic | cage | adult | JX090005 | N/S |
| C13d | Adriatic | cage | adult | JX090008 | JX090069 |
| C14a | Adriatic | cage | adult | N/S | JX090062 |
| C17a | Adriatic | cage | adult | JX089996 | N/S |
| C17b | Adriatic | cage | adult | JX089997 | JX090061 |
| Cf1a | Adriatic | cage | fingerling | JX090018 | JX090078 |
| Cf1c | Adriatic | cage | fingerling | JX090030 | JX090090 |
| Cf2a | Adriatic | cage | fingerling | JX090019 | JX090079 |
| Cf2b | Adriatic | cage | fingerling | JX090027 | JX090087 |
| Cf2c | Adriatic | cage | fingerling | JX090021 | JX090081 |
| Cf2d | Adriatic | cage | fingerling | JX090029 | JX090089 |
| Cf3a | Adriatic | cage | fingerling | JX090020 | JX090080 |
| Cf4a | Adriatic | cage | fingerling | JX090011 | JX090071 |
| Cf4b | Adriatic | cage | fingerling | JX090012 | JX090072 |
| Cf4c | Adriatic | cage | fingerling | JX090013 | JX090073 |
| Cf4d | Adriatic | cage | fingerling | JX090015 | JX090075 |
| Cf5a | Adriatic | cage | fingerling | JX090014 | JX090074 |
| Cf5b | Adriatic | cage | fingerling | JX090016 | JX090076 |
| Cf5c | Adriatic | cage | fingerling | JX090017 | JX090077 |
| Cf6b | Adriatic | cage | fingerling | JX090022 | JX090082 |
| Cf6c | Adriatic | cage | fingerling | JX090025 | JX090085 |
| Cf7a | Adriatic | cage | fingerling | JX090023 | JX090083 |
| Cf7b | Adriatic | cage | fingerling | JX090026 | JX090086 |
| Cf7c | Adriatic | cage | fingerling | JX090024 | JX090084 |
| Cf7d | Adriatic | cage | fingerling | JX090028 | JX090088 |
| W1a | Adriatic | wild | adult | JX090031 | JX090091 |
| W1b | Adriatic | wild | adult | JX090032 | JX090092 |
| W1c | Adriatic | wild | adult | JX090033 | JX090093 |
| W2 | Adriatic | wild | adult | JX090034 | JX090094 |
| W3 | Adriatic | wild | adult | JX090035 | JX090095 |
| W4a | Adriatic | wild | adult | JX090036 | JX090096 |
| W4b | Adriatic | wild | adult | JX090037 | JX090097 |
| W5a | Adriatic | wild | adult | JX090038 | JX090098 |
| W5b | Adriatic | wild | adult | JX090039 | JX090099 |
| W6a | Adriatic | wild | adult | JX090040 | N/S |
| W6b | Adriatic | wild | adult | JX090041 | N/S |
| W7 | Adriatic | wild | adult | JX090009 | N/S |
| W8 | Adriatic | wild | adult | JX090010 | N/S |
| W9 | Adriatic | wild | adult | N/S | JX090070 |
| FW1a | Gulf of Lion | wild | adult | JX089998 | JX090047 |
| FW1b | Gulf of Lion | wild | adult | JX089999 | JX090048 |
| FW2 | Gulf of Lion | wild | adult | JX090000 | JX090049 |
| FW3a | Gulf of Lion | wild | adult | N/S | JX090045 |
| FW3b | Gulf of Lion | wild | adult | N/S | JX090050 |
| FW4 | Gulf of Lion | wild | adult | N/S | JX090046 |
| FW5a | Gulf of Lion | wild | adult | N/S | JX090051 |
| FW5b | Gulf of Lion | wild | adult | N/S | JX090052 |
| FW6 | Gulf of Lion | wild | adult | N/S | JX090053 |
| FW7a | Gulf of Lion | wild | adult | N/S | JX090054 |
| C - cage, Cf - cage fingerlings; W - wild; FW - France wild, N/S - not sequenced. | | | | | |
