# Supplementary material for: Morphological Plasticity and Phylogeny in a Monogenean Parasite Transferring between Wild and Reared Fish Populations
Source: PLoS One. 2013 Apr 19;8(4):e62011. doi: 10.1371/journal.pone.0062011 (PMC3631154; doi:10.1371/journal.pone.0062011)
Supplement: Results S1 — Number of COI haplotypes obtained in each population and changes in base composition. (DOC) [file pone.0062011.s005.doc]

|  |  |  |  |  |  |  |
| --- | --- | --- | --- | --- | --- | --- |
|  |  |  |  |  |  | Pair base position |
| Haplotypes |  | Pop1 | Pop2 | Pop3 |  | (21)(26)(27)(41)(53)(65)(74)(131)(149)(164)(166) |
|  |  |  |  |  |  | (88)(195)(200)(203)(221)(242)(254)(268)(269) |
| Hap1 |  | 24 | 8 |  |  | GTTTTTCTGCGTTAATGTTG |
| Hap2 |  | 1 |  |  |  | ......T....C........ |
| Hap3 |  |  |  | 5 |  | ........AT....G.A.GT |
| Hap4 |  |  |  | 5 |  | .........T......A.GT |
| Hap5 |  |  |  | 5 |  | .......C.T....G.A... |
| Hap6 |  | 1 |  |  |  | .............G...... |
| Hap7 |  | 1 |  |  |  | .....C.............. |
| Hap8 |  | 3 | 1 |  |  | ............C....... |
| Hap9 |  |  | 1 |  |  | C................... |
| Hap10 |  |  | 1 |  |  | ..........T......... |
| Hap11 |  | 1 |  |  |  | ...C................ |
| Hap12 |  | 1 |  |  |  | ........A........... |
| Hap13 |  | 2 | 2 |  |  | .........T.......... |
| Hap14 |  | 1 |  |  |  | .C.............C.C.. |
| Hap15 |  | 1 |  |  |  | .....A...T.......... |
| Hap16 |  | 1 |  |  |  | ..C...T.......G.A... |
| Hap17 |  | 1 |  |  |  | ....C............... |
| total |  | 38 | 13 | 15 |  |  |
|  |  |  |  |  |  |  |
