# Supplementary material for: Morphological Plasticity and Phylogeny in a Monogenean Parasite Transferring between Wild and Reared Fish Populations
Source: PLoS One. 2013 Apr 19;8(4):e62011. doi: 10.1371/journal.pone.0062011 (PMC3631154; doi:10.1371/journal.pone.0062011)
Supplement: Results S2 — Number of ITS1 haplotypes obtained in each population with changes in base composition. (DOC) [file pone.0062011.s006.doc]

|  |  |  |  |  |  |  |
| --- | --- | --- | --- | --- | --- | --- |
|  |  |  |  |  |  | Pair base position |
| Haplotypes |  | Pop1 | Pop2 | Pop3 |  | (55)(56)(57)(98)(101)(103)(345)(346)(429)(457) |
|  |  |  |  |  |  | (497)(508)(541)(556)(577)(580)(734)(833)(847) |
| Hap1 |  | 30 | 8 | 6 |  | CACCCGAGGCGAGGGGAAG |
| Hap2 |  |  |  | 4 |  | AGTTA.............. |
| Hap3 |  | 1 |  |  |  | .......T........T.. |
| Hap4 |  | 1 |  |  |  | .......C........... |
| Hap5 |  | 1 |  |  |  | ........AGA.AAAA... |
| Hap6 |  | 1 |  |  |  | ......C............ |
| Hap7 |  | 1 |  |  |  | ...........T....... |
| Hap8 |  |  | 1 |  |  | .....T............. |
| Hap9 |  |  | 1 |  |  | .................TA |
| total |  | 35 | 10 | 10 |  |  |
|  |  |  |  |  |  |  |
