# Supplementary material for: Morphological Plasticity and Phylogeny in a Monogenean Parasite Transferring between Wild and Reared Fish Populations
Source: PLoS One. 2013 Apr 19;8(4):e62011. doi: 10.1371/journal.pone.0062011 (PMC3631154; doi:10.1371/journal.pone.0062011)
Supplement: Results S3 — Sampling localities, hosts and descriptive statistics of genetic diversity of Furnestinia echeneis , based on COI sequence data. (DOC) [file pone.0062011.s007.doc]

|  |  |  |  |  |  |  |  |  |  |  |  |  |  |  |  |  |
| --- | --- | --- | --- | --- | --- | --- | --- | --- | --- | --- | --- | --- | --- | --- | --- | --- |
| Site and sampling  year |  | Host |  | Pop |  | N |  | H |  | S |  | h |  | π |  | k |
| Adriatic Sea, 2010 |  | Cultured sea bream |  | Pop1 |  | 38 |  | 12 |  | 15 |  | 0.602 ± 0.093 |  | 0.0039 ± 0.0029 |  | 1.08393 ± 0.72923 |
| Adriatic Sea, 2010 |  | Wild sea bream |  | Pop2 |  | 13 |  | 5 |  | 4 |  | 0.628 ± 0.143 |  | 0.0026 ± 0.0024 |  | 0.74359 ± 0.58667 |
| Gulf of Lion, 2010 |  | Wild sea bream |  | Pop3 |  | 15 |  | 3 |  | 5 |  | 0.714 ± 0.046 |  | 0.0085 ± 0.0055 |  | 2.38095 ± 1.37151 |
| Total samples |  |  |  | Total |  | 66 |  | 17 |  | 20 |  | 0.749 ± 0.054 |  | 0.0088 ± 0.0011 |  | 2.4545 ± 1.34519 |
| N, sample size; H, number of haplotypes; S, number of segregating sites; h, haplotype diversity (±SD); π, nucleotide diversity (±SD); k, mean pairwise difference (±SD). | | | | | | | | | | | | | | | | |
