# Supplementary material for: Morphological Plasticity and Phylogeny in a Monogenean Parasite Transferring between Wild and Reared Fish Populations
Source: PLoS One. 2013 Apr 19;8(4):e62011. doi: 10.1371/journal.pone.0062011 (PMC3631154; doi:10.1371/journal.pone.0062011)
Supplement: Results S4 — Sampling localities, hosts and descriptive statistics of genetic diversity of Furnestinia echeneis , based on ITS1 sequence data. (DOC) [file pone.0062011.s008.doc]

|  |  |  |  |  |  |  |  |  |  |  |  |  |  |  |  |  |
| --- | --- | --- | --- | --- | --- | --- | --- | --- | --- | --- | --- | --- | --- | --- | --- | --- |
| Site and sampling  year |  | Host |  | Pop |  | N |  | H |  | S |  | h |  | π |  | k |
| Adriatic Sea, 2010 |  | Cultured sea bream |  | Pop1 |  | 35 |  | 6 |  | 11 |  | 0.269 ± 0.098 |  | 0.0008 ± 0.0007 |  | 0.6865 ± 0.53490 |
| Adriatic Sea, 2010 |  | Wild sea bream |  | Pop2 |  | 10 |  | 3 |  | 3 |  | 0.378 ± 0.181 |  | 0.0007 ± 0.0004 |  | 0.6009 ± 0.51979 |
| Gulf of Lion, 2010 |  | Wild sea bream |  | Pop3 |  | 10 |  | 2 |  | 5 |  | 0.533 ± 0.095 |  | 0.0031 ± 0.0022 |  | 2.6770 ± 1.55438 |
| Total samples |  |  |  | Total |  | 55 |  | 9 |  | 19 |  | 0.359 ± 0.082 |  | 0.0014 ± 0.0005 |  | 1.2365 ± 0.79618 |
| N, sample size; H, number of haplotypes; S, number of segregating sites; h, haplotype diversity (±SD); π, nucleotide diversity (±SD); k, mean pairwise difference (±SD). | | | | | | | | | | | | | | | | |
