# Supplementary material for: Morphological Plasticity and Phylogeny in a Monogenean Parasite Transferring between Wild and Reared Fish Populations
Source: PLoS One. 2013 Apr 19;8(4):e62011. doi: 10.1371/journal.pone.0062011 (PMC3631154; doi:10.1371/journal.pone.0062011)
Supplement: Results S5 — Analysis of molecular variance (AMOVA) for Furnestinia echeneis populations, based on mitochondrial COI and ITS1 (in italic) sequences data. (DOC) [file pone.0062011.s009.doc]

|  | | | | | | | | | | | | |
| --- | --- | --- | --- | --- | --- | --- | --- | --- | --- | --- | --- | --- |
| Source of variation |  | d.f. |  | Sum of squares |  | Variance components |  | Percentage of variation |  | F_ST_ |  | P |
| Among populations |  | 2 |  | 38.59 |  | 0.97 |  | 59.92 |  | 0.59921 |  | 0.00 |
| Within populations |  | 63 |  | 41.18 |  | 0.65 |  | 40.08 |  |  |  |  |
| Total |  | 65 |  | 79.77 |  | 1.63 |  |  |  |  |  |  |
|  |  |  |  |  |  |  |  |  |  |  |  |  |
| Among populations |  | 2 |  | 6.96 |  | 0.20 |  | 28.68 |  | 0.28679 |  | 0.00 |
| Within populations |  | 52 |  | 26.42 |  | 0.51 |  | 71.32 |  |  |  |  |
| Total |  | 54 |  | 33.38 |  | 0.71 |  |  |  |  |  |  |
|  | | | | | | | | | | | | |
