# Supplementary material for: Morphological Plasticity and Phylogeny in a Monogenean Parasite Transferring between Wild and Reared Fish Populations
Source: PLoS One. 2013 Apr 19;8(4):e62011. doi: 10.1371/journal.pone.0062011 (PMC3631154; doi:10.1371/journal.pone.0062011)
Supplement: Results S6 — Tajima's D , Fu's Fs statistics, corresponding P values and mismatch distribution parameter estimates for Furnestinia echeneis based on COI sequence data. (DOC) [file pone.0062011.s010.doc]

|  |  |  | | |  |  | | |  |  | | | | |  |  | | | | | | |
| --- | --- | --- | --- | --- | --- | --- | --- | --- | --- | --- | --- | --- | --- | --- | --- | --- | --- | --- | --- | --- | --- | --- |
|  |  | Tajima’s D | | |  | Fu’s Fs | | |  | Mismatch distribution | | | | |  | Goodness-of-fit tests | | | | | | |
|  |  | D |  | P |  | Fs |  | P |  | τ |  | θ_0_ |  | θ_1_ |  | SSD |  | P |  | HRI |  | P |
| Pop1 |  | -2.2407 |  | 0.0010 |  | -8.7983 |  | 0.0000 |  | 0.4453 |  | 0.5730 |  | 99999.0000 |  | 0.0022 |  | 0.7780 |  | 0.0559 |  | 0.8550 |
| Pop2 |  | -1.4371 |  | 0.0950 |  | -2.5349 |  | 0.0030 |  | 0.9394 |  | 0.0000 |  | 99999.0000 |  | 0.0248 |  | 0.2300 |  | 0.1911 |  | 0.1940 |
| Pop3 |  | 1.8498 |  | 0.9600 |  | 3.2757 |  | 0.9500 |  | 4.4257 |  | 0.0000 |  | 7.4609 |  | 0.1846 |  | 0.0570 |  | 0.6485 |  | 0.0020 |
| Total |  | -1.2726 |  | 0.0760 |  | -6.5209 |  | 0.0070 |  | 5.4707 |  | 0.0035 |  | 3.1703 |  | 0.0238 |  | 0.4600 |  | 0.0682 |  | 0.5300 |
|  | | | | | | | | | | | | | | | | | | | | | | |
