# Supplementary material for: Morphological Plasticity and Phylogeny in a Monogenean Parasite Transferring between Wild and Reared Fish Populations
Source: PLoS One. 2013 Apr 19;8(4):e62011. doi: 10.1371/journal.pone.0062011 (PMC3631154; doi:10.1371/journal.pone.0062011)
Supplement: Results S7 — Tajima's D , Fu's Fs statistics, corresponding P values and mismatch distribution parameter estimates for Furnestinia echeneis based on ITS1 sequence data. (DOC) [file pone.0062011.s011.doc]

|  |  |  | | |  |  | | |  |  | | | | |  |  | | | | | | |
| --- | --- | --- | --- | --- | --- | --- | --- | --- | --- | --- | --- | --- | --- | --- | --- | --- | --- | --- | --- | --- | --- | --- |
|  |  | Tajima’s D | | |  | Fu’s Fs | | |  | Mismatch distribution | | | | |  | Goodness-of-fit tests | | | | | | |
|  |  | D |  | P |  | Fs |  | P |  | τ |  | θ_0_ |  | θ_1_ |  | SSD |  | P |  | HRI |  | P |
| Pop1 |  | -2.3178 |  | 0.0010 |  | -1.6395 |  | 0.1280 |  | 3.0000 |  | 0.0000 |  | 0.2693 |  | 0.0513 |  | 0.0600 |  | 0.5791 |  | 0.5000 |
| Pop2 |  | -1.5622 |  | 0.0400 |  | 0.3901 |  | 0.5230 |  | 0.0000 |  | 0.0000 |  | 99999.0000 |  | 0.2143 |  | 0.0000 |  | 0.4592 |  | 0.9300 |
| Pop3 |  | 2.0401 |  | 0.9910 |  | 4.9515 |  | 0.9730 |  | 6.2070 |  | 0.0017 |  | 3.1752 |  | 0.3390 |  | 0.0400 |  | 0.7866 |  | 0.020 |
| Total |  | -2.1827 |  | 0.0010 |  | -2.1733 |  | 0.1370 |  | 3.0000 |  | 0.0000 |  | 0.4767 |  | 0.0711 |  | 0.1100 |  | 0.4618 |  | 0.5200 |
| SSD, sum of squared differences; HRI, Harpending's raggedness index. | | | | | | | | | | | | | | | | | | | | | | |
